# Simplifying the Measurement of College Students’ Career Planning: the Development of Career Student Planning Scale during the COVID-19 Pandemic

**DOI:** 10.1017/exp.2020.69

**Published:** 2021-01-18

**Authors:** Xiaoping Wang, Magnus A. Gray, Minsung Kim, Seungyeon Lee

**Affiliations:** 1 Second Xiangya Hospital, Department of psychiatry, 139 Renmin Middle Road, Changsha, Hunan, China, 410011; 1 School of Social & Behavioral Sciences, University of Arkansas at Monticello, 562 University Drive, Monticello, Arkansas 71656, USA; 2 Defense Language Institute Foreign Language Center, Presidio of Monterey, Monterey, CA93944, USA

**Keywords:** COVID-19 anxiety, career decision self-efficacy (CDSE), career student planning scale (CSPS), procrastination

## Abstract

We created a new, 8-item scale called “Career Student Planning Scale (CSPS)” for a valid and reliable measure regarding college students’ career planning during a traumatic event, such as a pandemic. CSPS is conceptually similar to the career decision-making difficulty questionnaire (CDDQ) and the career decision self-efficacy (CDSE) scale. CSPS leans towards questions about college students’ perceptions about career planning, rather than intuitions about career decision-making; it also inquires about how participants conceptualize about their career plans to be correct, rather than the more extreme idea about how their intuitions are correct: we developed this scale to capture the latter construct. We included the coronavirus anxiety scale (CAS), CDDQ, the general procrastination scale (GPS), and the CDSE short form (CDSE-SF) as covariates to ensure that CSPS has distinct effects on their career paths. Our findings indicate the CSPS has acceptable psychometric properties and demonstrates a valuable input to those measures.

## 1. Introduction

Given the extensive threats caused by the COVID-19 pandemic, college students’ mental health and career planning have become critical priorities across university campuses. Due to the novelty of this pandemic, little has been investigated on the relationship between coronavirus-related anxiety and young adults’ career decision making. Mahmud et al. (2020) found that fear of COVID-19 impacts career decisions through its inherent depression issues. This coincides with other empirical findings that show (a) young adults are more anxious about their careers, and (b) this anxiety has an impact on future career plans (Campagna & Curtis, 2007; Mojgan et al., 2011). Furthermore, Işik’s (2012) study showed there is a significantly negative relationship between career decision-making self-efficacy and trait anxiety. Together, these studies indicate that anxiety affects both young adults and college students with their careers, which is why we examine whether coronavirus anxiety affects both career planning and indecision. Career measures, like the career decision-making difficulty questionnaire (CDDQ) and the career decision-making self-efficacy scale (short form) (CDSE-SF) are widely used psychometric assessments, but how researchers quantify young adults’ attitudes regarding career planning has scarcely been explored (Gati et al., 1996; Gray et al., 2020; Lee et al., 2018; Tomaszek & Muchacka-Cymerman, 2020).

## 2. Objective

Psychometric assessment has an important role in health assessment and research. We created the 8-item college student planning scale (CSPS) to better quantify these variables. CSPS examines college students’ career plans to identify those affected with the pandemic’s uncertainty with most issues, including occupations and careers. We also investigated the degree to which CSPS relates to pre-existing and validated measures (i.e., the coronavirus anxiety scale [CAS], CDDQ, general procrastination scale [GPS], and the career decision self-efficacy scale [CDSE-SF]) during the recent pandemic. We examined the CSPS’s factorial structure and internal validity to better establish psychometric validation of the measure.

## 3. Methods

Participants (*N* =101) were originally recruited at small, liberal arts colleges in southeast Arkansas and southern California, except that one failed to answer some questions—which was excluded. Each participant voluntarily participated in the study, which was approved by the Institutional Review Board (IRB) at the University of Arkansas at Monticello and carried out with APA ethical standards. All participants received a copy of the consent form at the beginning of the study, and we provided debriefing after its completion. They completed the CSPS, CDDQ, GPS, and CDSE-SF in an online format and received extra credit for their involvement. The order of each questionnaire was randomized per participant to avoid an order effect. One-hundred participants were included for data analysis (*M* = 22.25, *SD* = 6.6; 77 females and 22 males). There were 25 Black (or African-American), 67 White, and 7 identifying with “others.” Four were listed as college freshmen, 34 were sophomores, 26 were juniors, and 33 were seniors enrolled full-time. Two listed having post-baccalaureate degrees.

A simple regression analysis was conducted to see how CSPS relates to CDDQ and CDSE-SF. The coefficient alpha of CSPS was 0.85, which is a desirable value as a measure, according to Cortina (1993). The CSPS’ significant correlation coefficients used solid planning (*r* = .29, p < .01) and management (*r* = .28, p < .01) showing potential validity evidence of its content, whether it is valid to the extent of planning management. After assessing reliability and validity of the CSPS, a regression analysis was conducted for the impact of CAS on CSPS. Two simple regressions of the CSPS were conducted to see the impact of the CDDQ and CDSE-SF.

## 4. Results

In all analyses, data from the CSPS, CAS, CDDQ, GPS, and CDSE-SF were used as continuous variables. Correlation coefficients among variables are shown in Table 1. CAS consists of 5 items, so we assume the measure has a tendency to assess anxiety related to COVID-19. Results of the first simple linear regression indicates that the effect between the CAS and CSPS was insignificant. The skewness value, 3.24, indicates a positively skewed response trend. Significant findings were assessed when examining the CSPS’s correlations with CDDQ, GPS, and CDSE (see Table 1).

**Table 1.** Correlations between CSPS, CDSE-SF, GPS, and CAS

	CSPS	CDSE-SF	GPS	CDDQ
CDSE-SF	0.52***			
GPS	-0.27**	-0.29**		
CDDQ	-0.47***	-0.33***	0.12	
CAS	-0.07	-0.06	0.04	0.16

p < .001 ‘***’, p < .01 ‘**’, p < .05 ‘*’

We also examined the factor structure among the given measures. Table 2 shows the item-level descriptive scale and internal consistency estimates among CSPS, CDSE-SF, GPS, CDDQ, and CAS. In the two regression analyses for CSPS, the new measure had a strong impact on CDDQ and CDSE-SF. The first CSPS simple linear regression indicated a significant effect on CDSE-SF, (F(1, 98) = 37.19, p < .001, R^2^ = .28) and how CSPS was a strong predictor (t = 6.1, p < .001) of the model. The second CSPS-SF simple linear regression concluded there was a similarly significant effect on CDDQ, (F(1, 98) = 27.67, p < .001, R^2^ = .22) and that CSPS was also a significant predictor (t = -5.3, p < .001) of the model.

**Table 2.** Internal consistency estimates of CSPS, CDSE-SF, GPS, CDDQ, and CAS

	No. of items	Coefficient alpha	Item scale
CSPS	8	0.85	1-5
CDSE-SF	25	0.95	1-5
GPS	20	0.79	1-5
CDDQ	32	0.93	1-9
CAS	5	0.96	0-4

## 5. Discussion

The current study examined the correlation between CSPS, CAS, CDDQ, GPS, and CDSE-SF. We first examined how COVID-19 anxiety affects college students and career planning; however, no correlation was shown between them. The results suggest that CAS is case-sensitive, so we caution against its use as a measure of anxiety for the general population. The CAS was newly developed in April 2020 when COVID-19 peaked and participants were a bit older (Lee, 2020). Previous literature shows a relationship between anxiety and career indecision (Campagna & Curtis, 2007; Işik, 2012; Mojgan et al., 2011; Tomaszek & Muchacka-Cymerman, 2020), so future research must focus on the correlation between generalized anxiety and career planning. More independent studies that investigate the psychometric properties of the CSPS are also necessary to establish the scientific rigor of this research.

No significant relationship was found between the CAS and CSPS, but our findings suggest moderate-sized correlations between CSPS-SF and CDDQ, and GPS and CDSE. A significant negative correlation was found between CSPS and CDDQ (*r* = -.47, p < .001). This suggests that college students will face fewer career difficulties if they plan for their future careers, possibly with some backup vocations. We found that the CSPS is negatively correlated with the GPS (*r* = -.27, p < .001), suggesting that factors related to procrastination may instigate career planning as well as decision difficulties. A significant positive relationship was found between the CSPS and CDSE-SF (*r* = .52, p < .001), indicating how adequate planning for a future career may allow college students to feel more confident about this decision. Overall, our study shows that CSPS can be a reliable, unidimensional construct for career planning in university samples, along with the general population.

## 6. Conclusion and Future Directions

This study serves as a psychometric analysis of the CSPS during the COVID-19 pandemic. Coronavirus-related anxiety has been a serious issue affecting young adults’ career planning and decisions, so we attempted to assess if COVID-19 anxiety affects the relationship between CSPS, CDDQ, GPS, and CDSE-SF among U.S. college students. The CAS was created for researchers and health professionals to identify anxiety and uncertainty in this growing pandemic, so the quality of our measures will hopefully provide solid evidence of how these measures were in fact examined, helping researchers select the best assessment plan.

One possible explanation about not finding a significant relationship between CAS and CSPS could be that young adults do not have an excessive fear of death, on which the 5 items of CAS specifically focus. All items ask about one’s physiologically-based anxiety reaction to COVID-19-related symptoms, which many young adults may not experience. Future studies will examine generalized anxiety with these measures about whether generalized anxiety is predictive of the actual population, rather than their current involvement with the recent pandemic.

We found interesting relationships between the CSPS and other measures in this study (i.e., the CDDQ, GPS, and CDSE-SF). In conclusion, the CSPS is a promising measure with relevance to college students. The higher scores of the CDSE correspond to higher levels of CSPS, which may contribute to growing mental symptoms, suggesting that without proper planning, this can lead to distraction in daily situations, such as college learning.

The current study represents a measurement of career planning, which will help us conduct future research related to college students’ mental health and professional development, with a mediation-based personality theory and career framework. The assessment of psychometric properties promotes the selection of valid and reliable instruments, so that researchers can ensure the internal validity of their results. An empirical investigation on how COVID-19-related anxiety impacts college students and their career plans should contribute to the current literature and to future researchers involved with the COVID-19 pandemic.

## Data Availability

Data for the experiments that is reported here is available upon request. The experiment was not preregistered.
